# Solitary osteochondroma of the twelfth rib with intraspinal extension and cord compression in a middle-aged patient

**DOI:** 10.1186/1471-2474-13-57

**Published:** 2012-04-13

**Authors:** Jung Hyun Shim, Choon Keun Park, Seung Ho Shin, Hee Sun Jeong, Jang Hoe Hwang

**Affiliations:** 1 Department of Neurosurgery, The Leon Wiltse Memorial Hospital, Suwon, Korea; 2 Department of Radiology, The Leon Wiltse Memorial Hospital, Suwon, Korea

## Abstract

**Background:**

Osteochondroma is a disease of growing bone and thus typically presents in younger patients. It has rarely been described in middle-aged and elderly patients. Data on the occurrence of osteochondroma show that the reported incidence of costal osteochondroma is very low. Moreover, costal osteochondroma arising at the costovertebral junction with neural foraminal extension and spinal cord compression is extremely rare.

**Case presentation:**

This study reports the case of a 58-year-old patient with a solitary osteochondroma of the 12th rib with intraspinal extension and spinal cord compression. The clinical history, plain radiographs, computed tomography (CT), magnetic resonance imaging, and pathologic findings of the reported patient have been reviewed. The relevant medical literature has also been reviewed. The patient was treated with surgery for complete tumour excision to avoid tumour recurrence. After surgery, the patient's symptoms improved. An additional CT scan obtained at 1 year after surgery did not show any evidence of recurrence.

**Conclusions:**

This patient is the oldest patient reported to have this rare form of costal osteochondroma. The age of the patient and the erosion of the adjacent bones raised clinical suspicion of malignancy; therefore, surgical management involved complete tumour excision with thoracolumbar fixation and fusion.

## Background

Osteochondromas are the most common benign tumours of the bone and may present as either a solitary lesion or as multiple lesions [1,2]. Solitary lesions are most common, but presentations of multiple lesions, usually with autosomal dominant inheritance, are termed hereditary multiple exostoses (HMEs) [1]. These tumours commonly occur in the long bones, but seldom affect ribs [3,4]. Only 1.5% of all osteochondromas are costal osteochondromas, and spinal cord compression due to a tumour arising from the head of a rib is even more rare [3,4].

We report a case that is unusual in many aspects such as the site of tumour origin within the rib; associated myelopathic symptoms; occurrence in a middle-aged patient; and the erosion and fusion of the vertebral pedicle, facet, and body due to tumour extension. Extension of the tumour necessitated total pediculectomy of T12, total facetectomy of T12-L1, and partial unilateral vertebrectomy of T12 with thoracolumbar fixation and fusion.

## Case presentation

A 58-year-old man presented with a 3-month history of right lower extremity weakness and numbness. He also reported progressive gait disturbance of 1-month duration. He denied bowel and bladder incontinence and sexual dysfunction. There was no medical or family history of neurologic dysfunction or bony tumour. Neurologic examination showed profound weakness, decreased pinprick sensation, and hyperreflexia of his right lower extremity. Right ankle clonus and positive Babinski response were also elicited.

**Figure 1:**
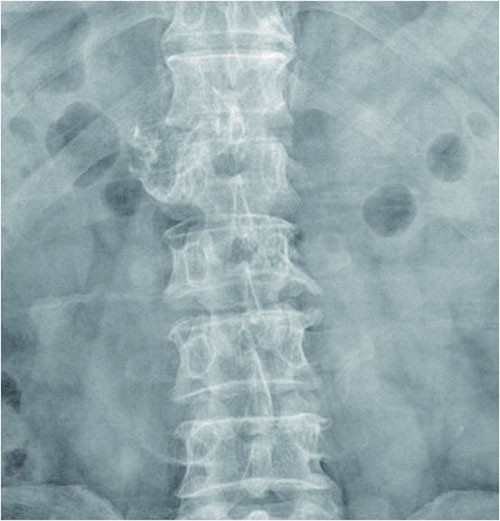
**Anteroposterior thoracolumbar plain radiograph**. The radiograph shows osseous exostoses on the right side of T12-L1.

**Figure 2:**
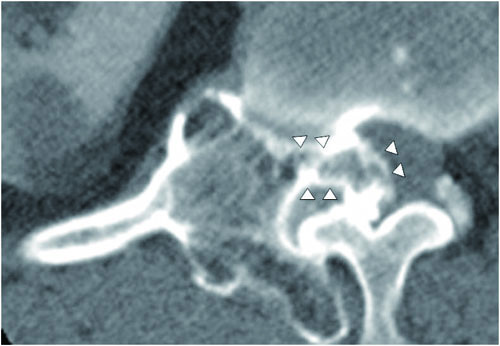
**Axial CT images**. The images show an osseous mass arising from the head of the right 12th rib at the costovertebral junction with obliteration of the neural foramen and intraspinal extension.

**Figure 3:**
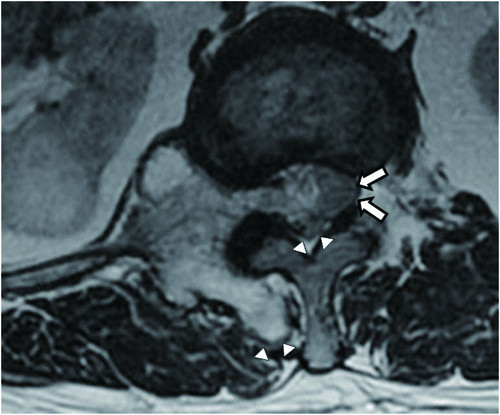
**Axial T2-weighted MR image**. The image shows an intraspinal osseous mass causing compressive myelopathy (arrows). Note 2 areas of the thin cartilaginous cap (arrowheads) on both the posterior and the intraspinal aspects of the mass.

Plain radiographs of the lower thoracic and lumbar spine regions showed an exophytic bony mass on the right side of T12-L1 (Figure 1). CT scans showed a multilobulated and sharply outlined osseous mass that was continuous with the cortical lines and the medulla of the head of the right 12th rib at the costovertebral junction (i.e. originating from the right 12th rib). The mass obliterated the neural foramen of T12-L1 and extended into the spinal canal, causing spinal cord compression. Pressure erosions, which suggested slow growth of the mass, were noted in the adjacent bony structures such as the vertebral pedicle and body. The pedicle of T12 showed bony fusion with the osseous mass, which was also suggestive of a slow-growing tumour. The cartilage cap, the pathognomonic sign of osteochondroma, was not well delineated on CT scans (Figure 2). Magnetic resonance (MR) imaging showed an extradural osseous mass arising from the right 12th rib with spinal cord compression and compressive myelopathy. A cartilage cap showing intermediate signal intensity on T1-weighted images and high signal intensity on T2-weighted images was observed on both the posterior and the intraspinal aspects of the osseous mass (Figure 3). The differential diagnosis of this lesion included chondrosarcoma. The presence of a thin cartilage cap (thickness < 1 cm) and the absence of any adjacent soft tissue mass formation strongly implied a benign tumour. However, the possibility of malignancy, such as chondrosarcoma, could not be definitively excluded because of the age of the patient and the presence of adjacent bone erosion. Therefore, en bloc surgery was selected as the treatment of choice.

**Figure 4:**
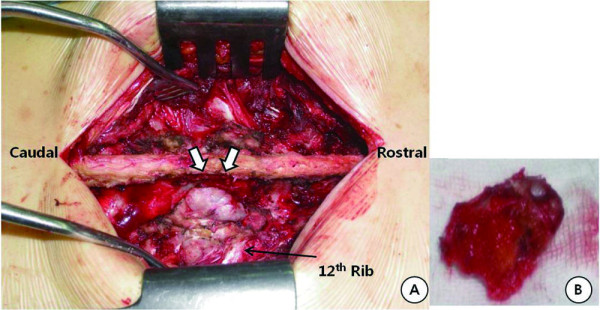
**A: Intraoperative photograph of the tumour before its resection**. A cartilaginous cap is evident (arrows). **B: **Macroscopic appearance of the tumour, with cartilaginous and osseous components.

A posterior midline skin incision was used to expose the posterior elements of T11 to L1, and the exposure was extended to both ribs. After dissection, the large outer fragment of the multilobulated osseous mass was completely exposed (Figure 4). This fragment was resected en bloc, resulting in a residual osseous mass originating from the right 12th rib and extending through the T12-L1 foramen to the spinal canal and the adjacent bony structures (i.e. vertebral pedicle and body). For safe excision of the residual fragment, the following procedures were performed sequentially using an air drill, allowing complete excision of the residual tumour mass: hemilaminectomy of the right T12 vertebra, total facetectomy of the right T12-L1 joint, partial removal of the proximal portion of the right 12th rib, total pediculectomy and partial vertebrectomy on the right side of T12 after exposure of the tumour in the canal, transection of the L1 transverse process, and resection of the tumour below the transverse process. On the left side, transpedicular screws and a rod were inserted into the T11-L1 vertebrae under C-arm fluoroscopic guidance. Laminar decortications were performed on the left side of the T11, T12, and L1 vertebrae. After autologous iliac bone harvest was performed on the right side, autograft bone chips were placed on the decorticated laminae.

**Figure 5:**
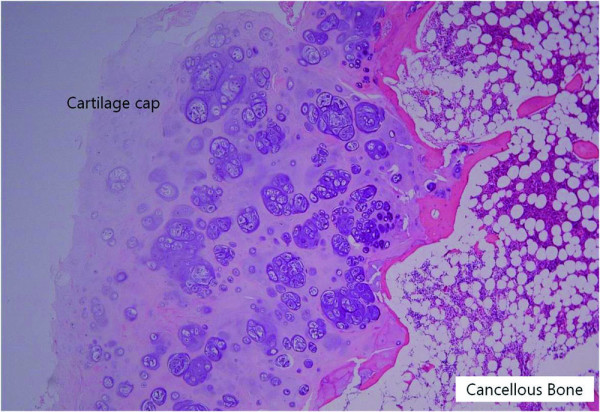
**Photomicrograph of the tissue specimen**. A cartilage cap covering the cancellous bone is observed. Trabecular bone and lipomatous marrow spaces are easily recognised on the right side. There were abundant clones of chondrocytes without nuclear atypia. Hematoxylin and eosin (H & E) stain; original magnification, 20×.

**Figure 6:**
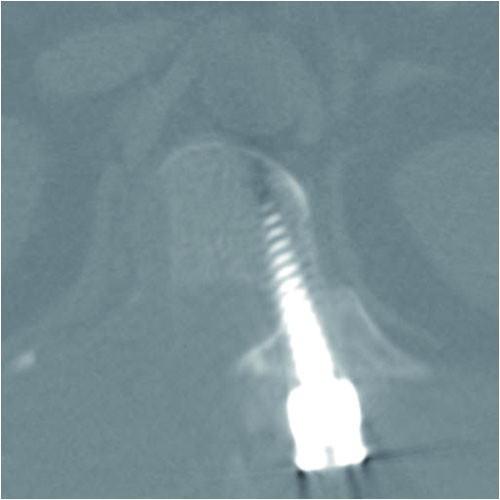
**Radiographs at 1-year follow-up**. Axial CT scan at the level of the T12 pedicle showing complete bony fusion and no evidence of recurrence.

Pathologic examination of the tissues of the lesion showed proliferated hyaline cartilage but atypical changes indicative of myxoid degeneration were not observed. The histologic examination results indicated the strong possibility of osteochondroma, but low-grade chondrosarcoma could not be excluded because of the age of the patient and the erosion of adjacent bones (Figure 5). After surgery, lower extremity weakness and numbness improved. One week after surgery, MR images and CT scans showed an enlarged spinal cord and complete resection of the osseous mass. At the 3-month follow-up examination, the weakness had completely resolved and the patient could ambulate independently. An additional CT scan obtained at 1 year after surgery did not show any evidence of tumour recurrence (Figure 6).

## Discussion

The reported incidence of costal osteochondroma is very low [3,4]. However, the actual incidence is likely to be underestimated because costal osteochondromas almost always arise at or near the costochondral junction and are usually asymptomatic [3-5]. The type of osteochondroma described in this case, arising at the costovertebral junction with neural foraminal extension and spinal cord compression, is extremely rare. Only 7 such cases have been previously reported in the English-language literature (Table 1) [3-8]. All those 7 patients were younger than 23 years (range, 12-23 years). Three were known to have HMEs, another 3 patients had solitary lesions, and no definitive data were available for the 7th patient. All the patients showed clinical improvement during the immediate follow-up period after complete tumour excision.

**Table 1:** Summary of the 7 previously reported cases of costal osteochondroma causing spinal cord compression

Age (yrs)	Sex	Rib of Origin	Approach Length of follow-up (months)		Outcome	Recurrence
[8] 12	M	5th	L	ND	CR	ND
[8] 11	F	4th	L	6	PR	ND
[7] 21	M	5th	T	6	CR	No
[6] 17	F	10th	L	3	CR	No
[4] 16	F	12th	L + F	19	CR	No
[5] 12	F	6th	L	ND	PR	ND
[3] 23	M	5th	L + F	6	CR	No

L: Laminectomy, T: Thoracotomy, F: Facetectomy, ND: No data available, CR: Completely recovered, PR: Partially recovered

Osteochondroma is a disease of growing bone, and thus typically presents in younger patients [9]. Tumour growth occurs early during childhood and usually arrests after puberty when the epiphysis closes [3]. Osteochondroma has rarely been described in middle-aged and elderly patients [10,11]. The pathophysiologic mechanisms underlying late-onset disease are not clearly understood. Some researchers believe that malignant transformation may abruptly increase the size of the lesion, thereby resulting in symptoms in older patients [12,13]. In the present case, the patient was 58 years old and is the oldest reported patient with costal osteochondroma. Careful histopathologic examination indicated the absence of malignancy.

Although the location of the bony portion of the osteochondroma can be reliably determined using multiplanar CT reconstruction, the exact size of the tumour may be underestimated because the cartilage cap of the tumour is not detectable by CT [3,4]. MR imaging is the best radiologic modality for evaluating the hyaline cartilage cap. The non-mineralized portions of the cartilage cap have high water content, resulting in intermediate-to-low signal intensity on T1-weighted images and high signal intensity on T2-weighted MR images. These features allow for accurate measurement of the thickness of the cartilage cap and distinction from overlying muscle on MR images [14]. In this case, the unique curvilinear high-signalintensity region covering the tumour, seen on axial T2-weighted images, represents the cartilage cap, which led to accurate preoperative diagnosis.

Malignant transformation, usually into a chondrosarcoma, occurs in approximately 1% of solitary osteochondromas and 10% of HMEs [9,12]. A sudden increase in lesion size or the development of new-onset pain suggests malignant transformation [12,13]. Bess et al. [15] emphasised that preoperative radiographic evaluation should consist of MR and CT imaging in order to provide optimal information about the lesion, which aids in surgical planning [3]. Radiologic findings may show consistent growth of exostoses after closure of the growth plate, alterations in surface delineation in comparison with previous radiographic studies, internal lytic areas, erosion or destruction of adjacent bones, and the presence of soft tissue masses containing scattered or irregular calcifications [15,16]. The size of the cartilaginous cap is the best indicator of malignancy [17]. MR imaging results showing a cartilage cap thickness exceeding 2 cm in adults and 3 cm in children should raise the suspicion of malignancy [18]. The use of gadolinium diethylenetriamine-pentacetate (Gd-DTPA)-enhanced MR imaging is an effective procedure for obtaining a differential diagnosis between malignant and benign lesions [19]. Generally, osteochondromas do not show contrast enhancement, but mild enhancement may be observed within the marrow [12]. In this case, Gd-DTPA-enhanced MR imaging was used, and the images did not show contrast enhancement. Because asymptomatic solitary osteochondromas have a low rate of malignant transformation, they can be followed up conservatively [20,21]. When the tumour causes pain or neurologic complications because of compression, or when the diagnosis is uncertain, the tumour should be completely excised to avoid tumour recurrence [22,23]. For any recurrence, the possibility of malignant transformation of the osteochondroma or of a low-grade chondrosarcoma that was initially poorly classified should be anticipated [24]. Because of the age of the patient and the presence of erosion into adjacent bones, the clinical suspicion for malignancy was high in this case. A surgical strategy for complete tumour excision was carefully planned to prevent tumour recurrence. As osteochondromas may pass through the neural foramen and lead to cord compression, the surgical approach should include decompression surgery such as laminectomy and/or facetectomy at the corresponding level [3]. If additional facetectomy is performed to remove the foramen and/or extraforaminal component, iatrogenic instability and kyphosis may occur during the follow-up period. In this case, hemilaminectomy of the right side of T12 and total facetectomy on the right side of T12-L1 provided wide exposure of the intraspinal and extraforaminal tumour originating from the rib. Additionally, because pressure erosions, suggestive of a slow-growing tumour, were noted in the adjacent bony structures, a total pediculectomy and partial vertebrectomy on the right side of T12 were performed, accompanied by the partial removal of the right proximal 12th rib. Therefore, it was essential to perform posterior thoracolumbar fixation and fusion for stability. However, fixation and fusion were performed only on the contralateral (left) side to avoid disturbing the radiologic follow-up required for monitoring tumour recurrence. Plain dynamic films showed that bone fusion was achieved 6 months after surgery. Although CT did not show any recurrence at the 1-year follow-up, further clinical and radiologic follow-up is required for monitoring tumour recurrence.

## Conclusions

The type of osteochondroma described in this case, which arose at the costovertebral junction with neural foraminal extension and spinal cord compression, is extremely rare. Only 7 such cases have been previously reported in the English-language literature. This is the first report of this form of rare costal osteochondroma occurring in a middle-aged patient and requiring surgical management involving complete tumour excision with thoracolumbar fixation and fusion. Malignancy remained a possibility throughout because of the patient's age and erosion of adjacent bones.

## Consent

Written informed consent was obtained from the patient for publication of this case report and any accompanying images. A copy of the written consent is available for review by the Editor-in-Chief of this journal.

## Competing interests

The authors declare that they have no competing interests.

## Authors' contributions

JH Shim provided major contributions to writing and editing the manuscript. CK Park supervised the writing of the manuscript. SH Shin contributed to analysis of the data. HS Jeong interpreted the radiologic results. JH Hwang contributed to conception and design of the data. All authors read and approved the final manuscript.

## Pre-publication history

The pre-publication history for this paper can be accessed here:

http://www.biomedcentral.com/1471-2474/13/57/prepub
